# Long-Range Correlations in Sentence Series from *A Story of the Stone*

**DOI:** 10.1371/journal.pone.0162423

**Published:** 2016-09-20

**Authors:** Tianguang Yang, Changgui Gu, Huijie Yang

**Affiliations:** 1 Department of Statistics, School of Mathematical Sciences, Nankai University, Tianjin 300071, P. R. China; 2 Department of Systems Science, Business School, University of Shanghai for Science and Technology, Shanghai 200093, P. R. China; Tianjin University, CHINA

## Abstract

A sentence is the natural unit of language. Patterns embedded in series of sentences can be used to model the formation and evolution of languages, and to solve practical problems such as evaluating linguistic ability. In this paper, we apply de-trended fluctuation analysis to detect long-range correlations embedded in sentence series from *A Story of the Stone*, one of the greatest masterpieces of Chinese literature. We identified a weak long-range correlation, with a Hurst exponent of 0.575±0.002 up to a scale of 10^4^. We used the structural stability to confirm the behavior of the long-range correlation, and found that different parts of the series had almost identical Hurst exponents. We found that noisy records can lead to false results and conclusions, even if the noise covers a limited proportion of the total records (e.g., less than 1%). Thus, the structural stability test is an essential procedure for confirming the existence of long-range correlations, which has been widely neglected in previous studies. Furthermore, a combination of de-trended fluctuation analysis and diffusion entropy analysis demonstrated that the sentence series was generated by a fractional Brownian motion.

## Introduction

Language is what makes us human [1, 2]. Over more than half a century, researchers have found rich patterns embedded in texts. For example, if we rank words in descending order of their frequencies, the frequency generally decreases according to a power-law of the rank, called Zipf’s law [3]. Additionally, there are long-range correlations in the use of words [4–7]; words reoccur according to a stretched exponential distribution [8, 9]; and network based approaches can expose interesting characteristics of the relationships between words (e.g., small-world properties) [10–12]. These findings can shed light on the dynamical mechanisms behind the formation and evolution of languages. They also provide quantitative measures of language characteristics that can be used in various practical problems, such as distinguishing different authors and monitoring the development of linguistic abilities.

Relevant literature has mainly focused on words, but sentences are the natural unit of a text. Sentences supply a context to restrict a word to an exact meaning, and are organized logically into sentence groups, paragraphs, chapters, and entire texts to express ideas at different levels. Rather than syntactical principles, the sequential order of sentences is a result of many interacting factors such as logic, fluency, rhythm, harmony, intonation, and the author’s style. A very recent paper by Drozdz et al. [13] investigated series of sentence lengths and found almost perfect long-range correlations in more than one hundred classical novels from around the world.

It is well known that Chinese language is significantly different to western languages. In Chinese language, an elementary meaning is usually expressed with a single character instead of a word, and sometimes several characters are integrated into a group to express a unique meaning. Several short sentences with different subjects can occur within a single long sentence, if there are logical relationships. This is strictly forbidden in English. At a macroscopic level, Chinese people also have unique habits in terms of how they think. Hence, detecting scaling behaviors embedded in Chinese sentence series is an interesting and nontrivial task, and thus is the focus of this work. The contributions of our work can be summarized as follows.

We used de-trended fluctuation analysis (DFA) [14] to detect long-range correlations in the text of *A Story of the Stone*, which is one of the four most popular classical Chinese novels. We define the length of a sentence as the number of characters contained in the sentence. There exists a weak long-range correlation up to a scale of 10^4^ (the Hurst exponent is 0.575 ± 0.002). The correlation structure is stable, i.e., different parts of the series have almost the same Hurst exponent. It is widely believed that this novel was written by two authors, i.e., the first to the 80th chapters were written by Xueqin Cao, and the 81th to the 120th chapters were written by E Gao, although there are still some disagreements [15]. However, we found no distinguishable difference in the long-range correlations of these two parts.

The 30th chapter of the novel uses the traditional Chinese punctuation symbol that represents a full stop (a small open circle), whereas the other chapters use the English full stop. This noise was used to test the impact of polluted data on long-range correlations detected using DFA. We found that introducing a small number of bad records (less than 1% of the total records) may result in incorrect conclusions. Therefore, to obtain reliable long-range correlation results we must test the structural stability of a series. To our knowledge, this procedure has been widely omitted in DFA calculations.

Furthermore, we combined DFA and diffusion entropy analysis (DEA) to find that the sentence series followed a fractional Brownian motion.

## Materials and Methods

### Data

*A Story of the Stone* is a masterpiece of Chinese vernacular literature and one of the four greatest Chinese classical novels [15]. It is remarkable not only for its huge cast of characters and psychological scope, but also for its precise and detailed observation of life and social structures in the 18th century China. It was written over approximately 10 years from 1749 to 1759. However, it was published anonymously until the 20th century, so there is some debate as to the author contributions. Currently, the 1st to 80th chapters are attributed to Xueqin Cao, and the remaining chapters are attributed to E Gao.

The text can be downloaded from the public repository *FigShare* with the accession number doi:10.6084/m9.figshare.3759300 (see also S1 File in the Supporting Information). From the text, we identified the positions of full stops (“.”), question marks (“?”), exclamation marks (“!”) and suspension points (“……”). Then, we calculated the increments from the successive positions to form the sentence length series, each element of which is regarded as the length of the corresponding sentence. The sentence length series for the whole text contains 34,759 elements. The two parts attributed to Xueqin Cao and E Gao, hereafter called X-part and E-part, are 23,504 and 11,255 elements long, respectively.

### Long-range correlations

Consider the sentence length series, *X* = {*x*_1_, *x*_2_, ⋯, *x*_*N*_}, where *x*_*n*_ is the length of the *n*th sentence, and *N* the total number of sentences. If *X* is stationary, a long-range power-law correlation implies that the autocorrelation function decays according to a power-law [16],

$$C\left(\tau \right)\equiv \frac{1}{N-\tau }\sum_{m=1}^{N-\tau }\frac{\left(x_{m}-<X>\right)\left(x_{m+\tau }-<X>\right)}{\sigma^{2}}∼\tau^{-\alpha },$$ (1)

where *σ* and <*X*> are the standard deviation and average of *X*, respectively.

The series *X* can be described using a random walk with displacements

$$y_{n}=\sum_{k=1}^{n}\left(x_{k}-<X>\right),n=1,2,\cdots ,N,$$ (2)

i.e., the *n*th displacement is the summation of all the first to *n*th elements in *X*. This integrated series is called the profile series, denoted by *Y*. Using the profile series, we can also define a set of displacements in a specified duration *τ*, Δ*y*(*τ*) = {*y*_1+*τ*_ − *y*_1_, *y*_2+*τ*_ − *y*_2_, ⋯, *y*_*N*_ − *y*_*N*−*τ*_}, and estimate the probability distribution function of Δ*y*, denoted as *p*(Δ*y*). If *X* has the long-range correlation defined in Eq (1), the profile series will be scale-invariant (self-similar) [17], namely,

$$p\left[\Delta y\left(\tau \right)\right]∼\frac{1}{\tau^{\delta }}F\left[\frac{\Delta y(\tau )}{\tau^{\delta }}\right],$$ (3)

where *δ* is the scaling exponent and *F*(.) a function. There exists a simple relation between *δ* and *α*, which is $\delta =1-\frac{\alpha }{2}$ [18, 19]. Hence, the scaling exponent *δ* can be used to measure long-range correlation behavior in the initial series, *X*. However, the sentence series (*X*) is generally non-stationary, so special procedures are required to evaluate the scaling exponent, *δ*.

#### De-trended fluctuation analysis

DFA is a widely used method for detecting long-range correlations embedded in non-stationary time series [14]. We briefly describe it, so that this paper is as self-contained as possible.

First, we extract all possible segments with a predefined length *w* (window size) from the profile series *Y*. That is,

$$Z_{n}=\left\{y_{n},y_{n+1},\cdots ,y_{n+w-1}\right\},n=1,2,\cdots ,N-w+1.$$ (4)

Second, we fit each segment with a *q* order polynomial function, $f\left(i,n\right)=\sum_{b=0}^{q}a_{b}\left(n\right)i^{b}$, where *a*_*b*_(*n*), *b* = 0, 1, ⋯, *q* are the fitting parameters for the *n*th segment, and *i* = 1, 2, ⋯, *w*. The fitting curves are taken as estimates of trends in the corresponding segments. Subtracting the trends from the profile segments results in a set of segments of a stationary series,

$$\begin{array}{c} S_{n}=\left\{y_{n}-f\left(1,n\right),y_{n+1}-f\left(2,n\right),\cdots ,y_{n+w-1}-f\left(w,n\right)\right\},\\ n=1,2,\cdots ,N-w+1. \end{array}$$ (5)

If there are long-range correlations, the standard deviation, *DFA*_*q*_, will obey a power-law,

$$DFA_{q}\left(w\right)\equiv \sqrt{\frac{\sum_{n=1}^{N-w+1}\sum_{k=1}^{w}{\left[y_{n+k-1}-f\left(k,n\right)\right]}^{2}}{w(N-w+1)}}∼w^{H}.$$ (6)

For completely random, persistent, and anti-persistent series, we will have *H* = 0.5, *H* > 0.5, and *H* < 0.5, respectively.

In our calculations, the order of the polynomial function was *q* = 2. Because we are interested in scaling behaviors, the scale ranges, for instance, of 10^0^–10^1^, 10^1^–10^2^, 10^2^–10^3^, and 10^3^–10^4^ should have the same contributions when estimating the Hurst exponent. The window sizes were *w* = [1.2^*m*^],*m* = 1, 2, ⋯, and $q+3\leq w\leq \frac{N}{3}$, where [⋅] is the integer part of a real number. If we plot *log*[*F*_*q*_(*w*)] versus *log*(*w*), the points are distributed at almost identical intervals along the *log*(*w*) axis. Accordingly, in the least square estimate of the Hurst exponent, all the scales contain almost the same number of points and consequently have identical contributions.

### Diffusion Entropy Analysis

Alternatively, the scaling exponent *δ* can be evaluated using diffusion entropy analysis (DEA) [20–22]. Starting from Eq (3), a straightforward computation leads to a simple relation of Shannon entropy versus the scaling exponent. That is,

$$E\left(\tau \right)\equiv -\int_{-\infty }^{+\infty }p\left[\Delta y\left(\tau \right)\right]lnp\left[\Delta y\left(\tau \right)\right]d\left[\Delta y\left(\tau \right)\right]∼A+\delta ln\tau .$$ (7)

Hence, the slope of this relation is an unbiased estimate of *δ* if the sentence length series is stationary, which is generally not the case in reality.

We used the central moving average method [23–26] to extract the trend in the displacement series, Δ*y*(*τ*). Let a window with size *τ* slide along this series. The average value of the covered displacements is regarded as the trend of the central displacement, namely, the $\left[\frac{\tau +1}{2}\right]$th displacement, where [⋅] is the integer part of a real number. Subtracting the trend from the displacement series results in a de-trended displacement series with length *N* − 2*τ* + 1.

We divided the interval so that the elements of the de-trended displacement series were distributed into *R*(*t*) bins, and calculated the number of de-trended displacements occurring in every bin, denoted as *r*(*τ*, *j*), *j* = 1, 2, ⋯, *R*(*τ*). Then, the probability distribution can be approximated using

$$p\left(\tau ,j\right)\approx \frac{r(\tau ,j)}{N-2\tau +1},j=1,2,\cdots ,R\left(\tau \right).$$ (8)

The Shannon entropy is then estimated using

$$DE\left(\tau \right)=-\sum_{j=1}^{R(\tau )}\frac{r(\tau ,j)}{N-2\tau +1}ln\left[\frac{r(\tau ,j)}{N-2\tau +1}\right].$$ (9)

If *DE*(*τ*) obeys the relation in Eq (7), the sentence length series is scale-invariant. This method is called diffusion entropy analysis (DEA).

### Joint use of DFA and DEA

DEA can produce unbiased evaluation of *δ* for any kind of process, whereas the Hurst exponent (*H*) obtained by DFA is dynamical process dependent (this is why we use *H* instead of *δ* to represent the calculated scaling exponent in the DFA method) [22]. For a fractional Brownian motion, the estimated value of *H* is unbiased, namely, *H* = *δ*. For a Levy walk process, we have a quantitative relation $\delta_{\text{levy}}=\frac{1}{3-2H}\leq H$. For a Levy flight process, the variance diverges and the DFA fails to qualitatively detect the scaling behavior. Hence, a combination of DEA and DFA (i.e., the relationship between *H* and *δ*) can provide information on the dynamic mechanism [22, 27–31].

## Results

**Fig 1:**
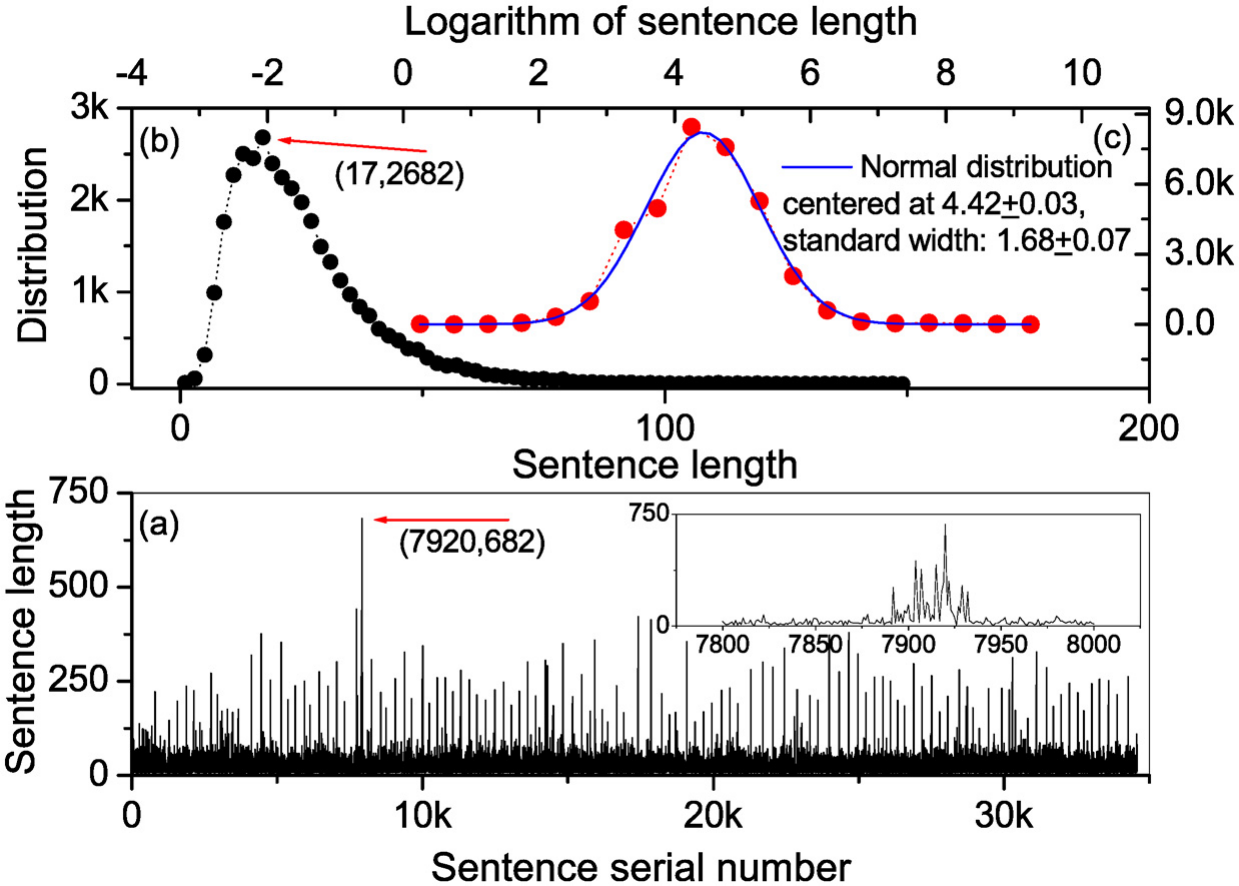
Statistics of sentence lengths. (a) Sentence length series. *Inset*: some sentences with large lengths are clustered together. This cluster is due to the traditional Chinese full stop symbol (small open circle) that was used in the 30th chapter, which produced noisy data. (b) Sentence length follows a right-skewed distribution, which is the log-normal distribution in (c).

In the traditional Chinese punctuation system, a full stop is represented by a small open circle. However, with the increased computer usage, the English language full stop (“.”) has found its way into the Chinese language punctuation system. As a result, the two symbols have become acceptable. In the original downloaded text, the small open circle was used in the 30th chapter, while the rest of the chapters were punctuated with the English full stop (“.”) to signify the end of a sentence. We therefore recognize the symbol “.” as a full stop, and the small open circle as an independent Chinese character. This way, noise was introduced in the sentence series leading to a wrong number of identified sentences in the 30th chapter, and contaminating about 1% of the sentence lengths in the whole novel. Fig 1(a) presents the sentence length series. Most of the sentences are short, while some sentences are tens to hundreds of characters long. As shown in the inset panel of (a), some long sentences are clustered at around the 7850–7950th elements. This clustering comes from polluted records in the 30th chapter.

The sentence length obeys a right-skewed distribution as shown by the black solid circles in Fig 1(b). This distribution is log-normal (see the red solid circles in Fig 1(c)), i.e., the logarithm of sentence length is normally distributed. The characteristic length is 17. This distribution can be understood in terms of stochastic models of language [32, 33]. Consider a sentence with length *L*, and for simplicity assume that every character appears in a certain position of the sentence with identical probability, namely, $\frac{1}{L}$. The quantity of information contained in the sentence can be measured using Shannon entropy, $-\sum_{m=1}^{L}\frac{1}{L}\times ln\left(\frac{1}{L}\right)=lnL$. The log-normal distribution in Fig 1(c) tells us that the information contained in the sentences is distributed according to a Gaussian function.

**Fig 2:**
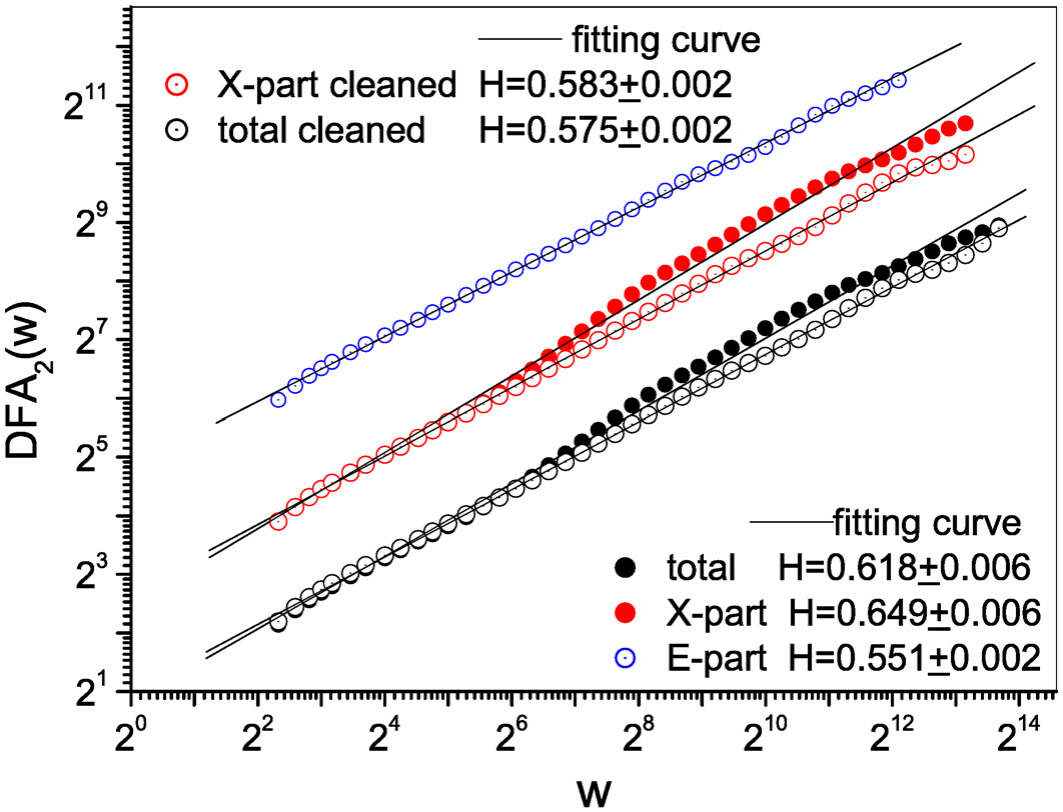
Long-range correlations in the noisy and cleaned series. Noisy records can result in incorrect estimates of the Hurst exponents (solid circles) and consequently false conclusions, even if they only cover a limited proportion of the total series. The effect of noise can be removed using a cleansing procedure (open circles). The X-and E-part of the text are the first to 80th chapters, and the 81th to the 120th chapters, which are currently attributed to Xueqin Cao and E Gao, respectively.

Using the noisy series (extracted from the original text), we calculated *DFA*_2_ for the total series, the series for the X-part (Chapters01–80), and the series for the E-part (Chapters81–120) as shown in Fig 2. The curves for the entire text and the X-part decrease slightly at large scales (black and red solid circles), but can be regarded as roughly obeying the power-law with Hurst exponents of *H* = 0.618 ± 0.006 and *H* = 0.649 ± 0.006, respectively. The E-part follows a perfect power-law relationship with a Hurst exponent of *H* = 0.55 ± 0.002.

This result seems to be perfect, because we can conclude that the whole text, the X-part, and the E-part all have long-range correlations over a wide range (10^1^–10^4^). Furthermore, the X-part has a significantly high Hurst exponent compared with that of the E-part (the difference was Δ*H* = 0.649 − 0.55 ≈ 0.1). This appears to be because the two parts were written by different authors. Thus, it appears that we can distinguish between the language of the two authors.

But is this true? To answer this question, we used a cleansing procedure to filter out the effects of the noise. We replaced all the small open circles in the 30th chapter with the full stop “.” and re-extracted the sentence series, called cleaned series. As shown in Fig 2, the noise induced deformations were exactly corrected. The Hurst exponents were 0.575, 0.585, and 0.551 for the cleaned total, cleaned X-part, and the E-part (not polluted) respectively. They are almost identical, and the X-part and E-part are no longer distinguishable. So the polluted records may lead to exciting but incorrect conclusions. Hence, one must be careful when using the DFA method to detect long-range correlations, even when using sufficiently long series.

However, using the sentence length series in Fig 1(a), we cannot find significant evidence for the noise. Actually, when we investigate empirical series, there are typically some state changes of the measured objects. For example, the physiological state of a volunteer may change occasionally in a long-term experiment, e.g., walking or sleeping. These occasional changes lead to abrupt changes in the records. But it is very difficult to find evidence of this sort of noise. Determining the correctness of the DFA calculations is non-trivial and cannot be solved using a single result for a series with a specific length. Herein, we will show that a structural stability test may help to confirm the existence of long-range correlations.

**Fig 3:**
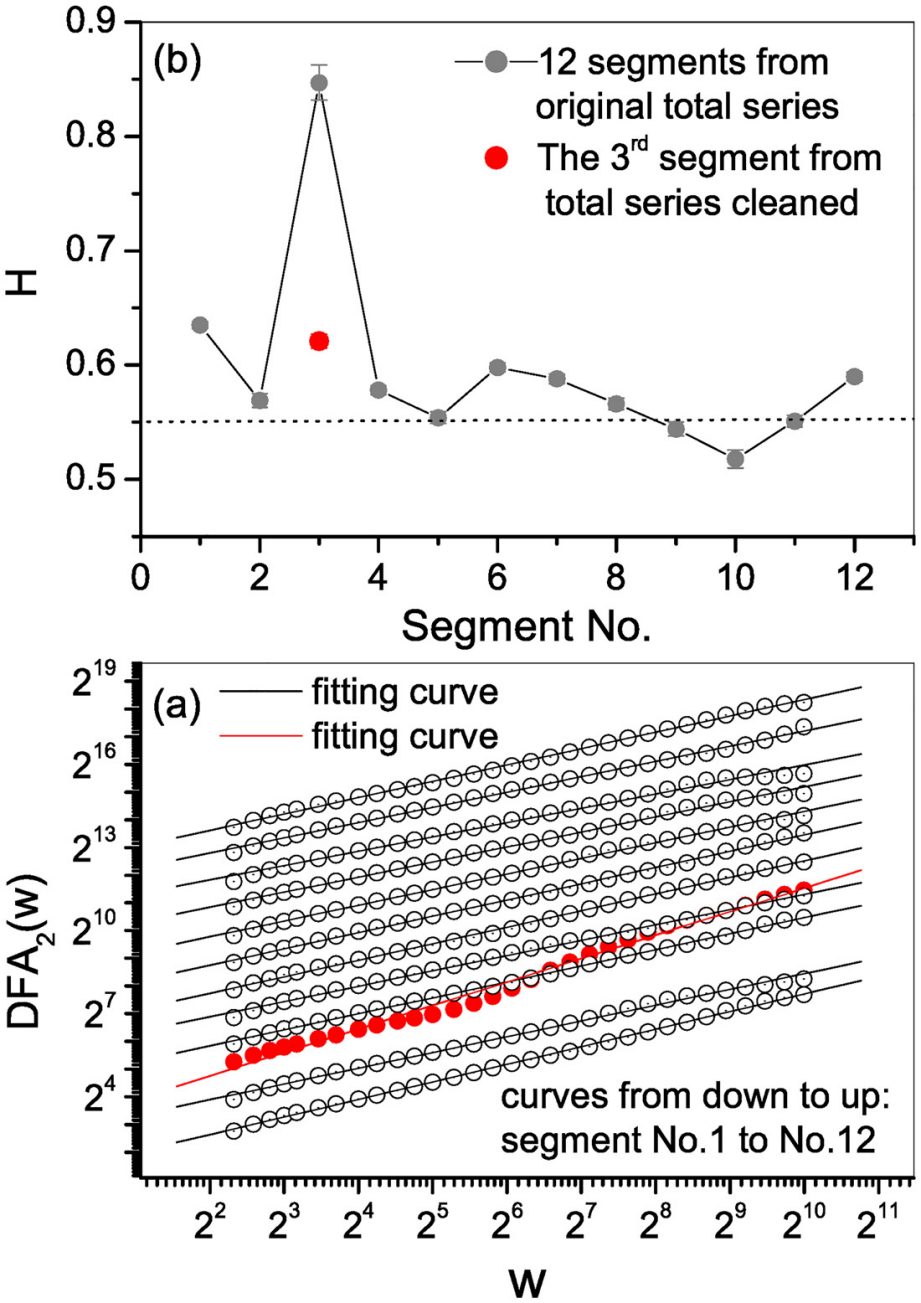
Structural stability test. The total polluted series was separated into 12 non-overlapping segments with a length of 2881. (a) All the curves obey almost perfect power-law relationships (gray open circles), except the 3rd segment which deviates significantly (red solid circles). (b) Hurst exponents for the 12 segments. The unreasonable large value of 0.87 for the 3rd noisy segment was corrected to 0.621 (red solid circle) after applying the cleaning procedure. There is a slightly decreasing trend.

We separated the noisy series into 12 non-overlapping segments that were 2881 characters long. The third segment covers the noisy records. For every segment, we calculated the *DFA*_2_ as shown in Fig 3(a). All the curves (gray open circles) almost exactly followed a power-law relationship, except for the 3rd segment (red solid circles), which significantly deviated from a straight line. A linear fit resulted in an unreasonably large estimated slope (0.87, see Fig 3(b)).

Using the cleaned series, there was a slightly decreasing trend in the scaling exponents. The unreasonable scaling exponent for the 3rd segment reduced to 0.621 (see Fig 3(b)—red solid circle), while the 8th to 12th segments exhibited comparatively smaller values. Since the novel was written over a long period (approximately 10 years), it is difficult to attribute this finding to differences in the two authors’ style or to a style change of a single author. Moreover, since most of the segments portray very similar scaling exponents, we conclude that they are structurally stable thus confirming the existence of long-range correlations in the whole series.

**Fig 4:**
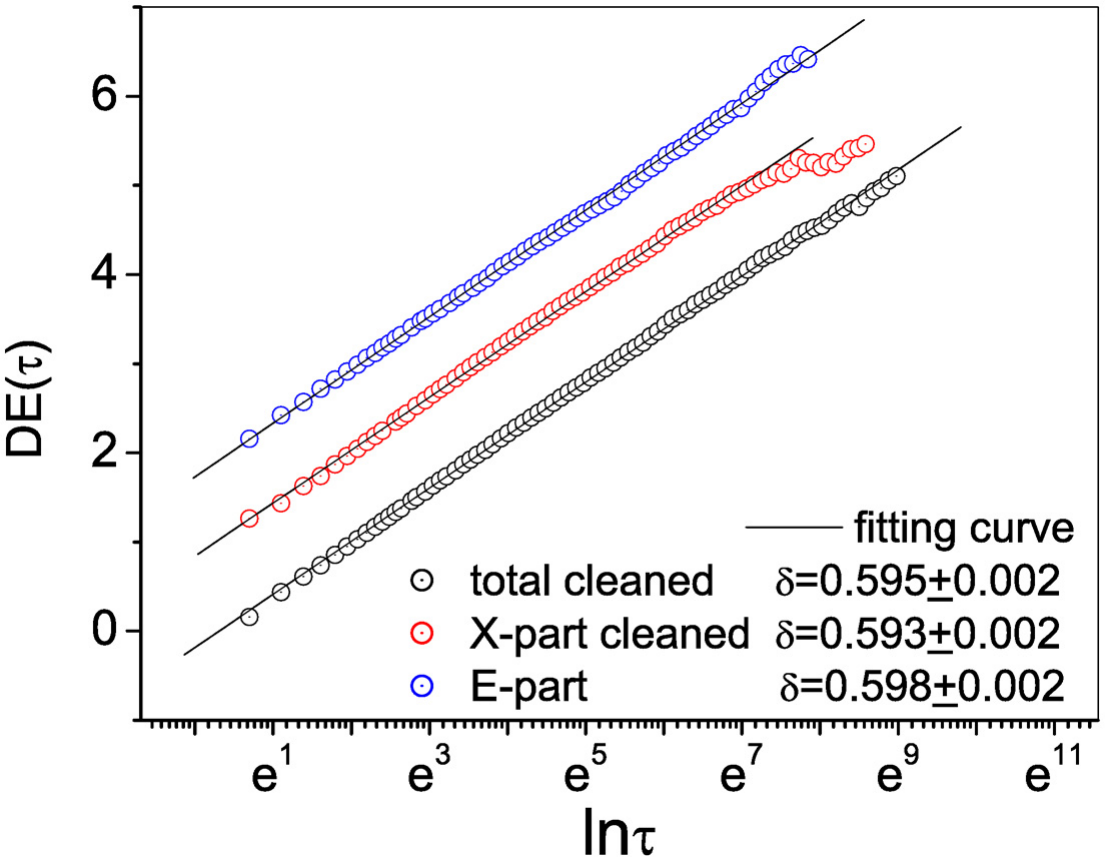
Diffusion entropy analysis of the cleaned total, cleaned X-part, and E-part series. Estimated values of the scaling exponent for the three series were almost identical.

Additionally, the DEA method was used to detect the scaling behaviors embedded in the cleaned total, cleaned X-part, and E-part series. Fig 4 contains plots of diffusion entropy to *lnτ* and the estimated values of the scaling-invariant exponent. For the total, X-part, and E-part series, the estimates were (*H*, *δ*) = (0.575, 0.595), (0.583, 0.593) and (0.551, 0.598), with corresponding values for $\frac{1}{3-2H}$ of 0.541, 0.545 and 0.527, respectively. Hence, we have $\frac{1}{3-2H}>H$ in all three cases. Because the error interval was [−0.002, +0.002], the results support the conclusion of *δ* = *H*. Consequently, the cleansed sentence series was generated by a fractional Brownian motion.

To ensure that the scaling behaviors were dependent on the sequential order of the sentences, we shuffled the sentence series and its segments and recalculated the scaling exponents. This resulted in *H* = 0.5 with confidence intervals less than [−0.003, +0.003] (not shown).

## Conclusion

In summary, non-trivial patterns in sentence length series can provide information on the structural behaviors of a text, which reflect characteristics of, for example, the author’s style and logic. This information can be used to construct theoretical models of language formation and evolution, and also be helpful to solve practical problems such as evaluating linguistic ability and distinguishing different authors. Unlike most western languages, the Chinese language differs with respect to its organization at different levels. This work thus focused on a Chinese famous novel, *A Story of the Stone*, which is attributed to two different authors over a span of ten years.

Weak long-range correlations of the text were found using the DFA and DEA methods. The scaling behavior was perfect over a long scale covering approximately 10^1^–10^4^. The scaling invariance was confirmed using the structural stability test. Although the Hurst exponent slightly decreased with the sequential number of segments of the whole sentence series, we could not attribute this to the difference between two authors or to changes to a single author’s style over ten years.

Results showed that when the DFA method is applied to noisy data, it can produce exciting but false results leading to incorrect conclusions. This was the case even if the noise affects a small proportion of the total series (e.g. 1%) and the series is sufficiently long (in this paper, ∼3 × 10^4^). To confirm the existence of a long-range correlation, we should also apply the structural stability test to determine if different parts of the series have similar scale invariance. This procedure was rarely used in existing publications.

Furthermore, combining DFA and DEA we found that the sentence series of *A Story of the Stone* was produced by a fractional Brownian motion.

The structural stability test requires powerful new tools to detect scaling behaviors from short time series (see, for example, [34–41]). To reach further conclusions on the Chinese language, we will investigate more Chinese novels in the future.

## Supporting Information

S1 File S1_File.txt. Cleaned text of the *A Story of the Stone*. The data can also be downloaded from the public repository *FigShare*, uploaded with a title of *A Story of the Stone* and an accession number of doi:10.6084/m9.figshare.3759300.
(TXT)

S2 File S2_File.m. Code for de-trended fluctuation analysis.
(M) Click here for additional data file.
